# Prevalence of hypertension (HT) among HIV infected patients from Galați

**DOI:** 10.1186/1471-2334-14-S4-P35

**Published:** 2014-05-29

**Authors:** Miruna Drăgănescu, Manuela Arbune

**Affiliations:** 1 “Dunărea de Jos” University, Galați, Romania

## 

With over 300 HIV infected patients, Galați is the fourth area of HIV prevalence, the majority is nosocomially infected being now young adults with old infection. Objectives: to determine the prevalence of hypertension (HT) and to compare the results with national references provided by SEPHAR study.

162 HIV positive patients 45% men, 55% women, usually assessed in Outpatient Clinic, aged from 20-54 years (mean 26 years) were included in this cross-sectional study. Data about blood pressure (BP, total and HDL-cholesterol CD4, weight, height, smoking were collected from patient dossier and compared with general population data. HT was defined according to ESH/ESC 2013 guidelines as systolic BP>140 mmHg or diastolic BP >90 mmHg. Statistical analysis: MedCalc.

Mean systolic BP (SBP) of HIV patients is 110±11.4 mmHg vs. 136±22.6 mmHg (p<0.0001). HT prevalence is 8% vs. 40% (p=0.0398) more frequent in HIV positive men (61.5%) than HIV positive women (38.5%). Abnormal total cholesterol is 32% vs. 26% (p=0.4407),HDL-cholesterol <55 mg% prevalence is 77% vs. 13% (p<0.0001). Smoking prevalence among HIV patients is 54.6% vs. 29% (p<0.0001) more frequent among men than women. Obesity by BMI is 1.23% for BMI>30, 15.4% for BMI 25-29. Mean CD4 is 698 cells/cmm. No correlation between CD4 levels and HT was made.

1. Overall HT appears to be rare among HIV population due probably to high prevalence of young age therefore further observation is required to establish the real prevalence among HIV patients. Men appear to be more affected by HT than women.

2. Dyslipidemia is more frequent among HIV patients than general population, low HDL-cholesterol prevalence is statistically significant higher than general population, as marker for chronic HIV infection.

3. There is no explanation for significant higher prevalence of smokers among HIV infected patients than general population; male gender is more affected than female gender.

4. Obesity by BMI is unusual among HIV patients due probably to young age and ongoing chronic infection.

5. In conclusion longitudinal study is needed for a better description of hypertension and cardiovascular risk factors among this special population.

